# The pseudokinase TRIB3 controls adipocyte lipid homeostasis and proliferation *in vitro* and *in vivo*

**DOI:** 10.1016/j.molmet.2023.101829

**Published:** 2023-10-30

**Authors:** Miguel Hernández-Quiles, Laura Martinez Campesino, Imogen Morris, Zabran Ilyas, Steve Reynolds, Nguan Soon Tan, Paula Sobrevals Alcaraz, Edwin C.A. Stigter, Ákos Varga, János Varga, Robert van Es, Harmjan Vos, Heather L. Wilson, Endre Kiss-Toth, Eric Kalkhoven

**Affiliations:** 1 Center for Molecular Medicine, University Medical Center Utrecht, Utrecht University, 3C584 CG Utrecht, The Netherlands; 2 Division of Clinical Medicine, School of Medicine and Population Health, University of Sheffield, Sheffield S10 2TN, UK; 3 Oncode Institute and Molecular Cancer Research, Center for Molecular Medicine, University Medical Center Utrecht, Utrecht University, 3C584 CG Utrecht, The Netherlands; 4 Department of Dermatology and Allergology, University of Szeged, H-6720 Szeged, Hungary; 5 Lee Kong Chian School of Medicine, Nanyang Technological University Singapore, Clinical Sciences Building, 11 Mandalay Road, 308232 Singapore, Singapore; 6 School of Biological Sciences, Nanyang Technological University Singapore, 60 Nanyang Drive, 637551 Singapore, Singapore

**Keywords:** Tribbles, Adipocyte, Omics analyses, Metabolism

## Abstract

**Objective:**

*In vivo* studies in humans and mice have implicated the pseudokinase Tribbles 3 (TRIB3) in various aspects of energy metabolism. Whilst cell-based studies indicate a role for TRIB3 in adipocyte differentiation and function, it is unclear if and how these cellular functions may contribute to overall metabolic health.

**Methods:**

We investigated the metabolic phenotype of whole-body *Trib3* knockout (*Trib3*^*KO*^) mice, focusing on adipocyte and adipose tissue functions. In addition, we combined lipidomics, transcriptomics, interactomics and phosphoproteomics analyses to elucidate cell-intrinsic functions of TRIB3 in pre- and mature adipocytes.

**Results:**

*Trib3*^*KO*^ mice display increased adiposity, but their insulin sensitivity remains unaltered. *Trib3*^KO^ adipocytes are smaller and display higher Proliferating Cell Nuclear Antigen (PCNA) levels, indicating potential alterations in either i) proliferation-differentiation balance, ii) impaired expansion after cell division, or iii) an altered balance between lipid storage and release, or a combination thereof. Lipidome analyses suggest TRIB3 involvement in the latter two processes, as triglyceride storage is reduced and membrane composition, which can restrain cellular expansion, is altered. Integrated interactome, phosphoproteome and transcriptome analyses support a role for TRIB3 in all three cellular processes through multiple cellular pathways, including Mitogen Activated Protein Kinase- (MAPK/ERK), Protein Kinase A (PKA)-mediated signaling and Transcription Factor 7 like 2 (TCF7L2) and Beta Catenin-mediated gene expression.

**Conclusions:**

Our findings support TRIB3 playing multiple distinct regulatory roles in the cytoplasm, nucleus and mitochondria, ultimately controlling adipose tissue homeostasis, rather than affecting a single cellular pathway.

## Highlights

• Adipose tissue TRIB3 expression alters with metabolic traits in mice and man.
• Trib3 depletion in mice gives increased adiposity but unaltered insulin sensitivity.
• Multiple-omics approaches implicate TRIB3 in adipose tissue homeostasis control.
• TRIB3 regulates the proliferation-differentiation balance and lipid handling in (pre)adipocytes.

## 1 Introduction

The prevalence of obesity and its related comorbidities have tripled since 1990 and the global incidence of type II diabetes is projected to reach 350 million cases by 2030 [1]. Obesity is a chronic, multifactorial disease, developed through the interaction between genetics and environmental factors, such as nutrition, physical activity and cultural influences [[2], [3], [4]]. The World Health Organization (WHO) defines obesity as an unhealthy state characterized by excessive and abnormal adiposity. This adipose alteration represents the first step into the development of chronic inflammation and insulin resistance, resulting in metabolic dysfunction [5]. Adipose tissue (AT) is responsible for the storage and release of free fatty acids in response to different metabolic needs as well as the regulation of whole-body metabolism through the production and secretion of adipose-specific chemokines [6,7]. Adipocyte differentiation and function are tightly controlled by a set of pro- and anti-adipogenic factors, such as peroxisome proliferator-activated receptor γ (PPARγ) and CCAAT/enhancer binding proteins (C/EBP) as pro-adipogenic factors [8] and Wnt signaling as an anti-adipogenic pathway [9,10]. Prolonged and excessive exposure to a high-caloric diet together with a sedentary lifestyle result in an increase in adipocyte number and size. These hypertrophic adipocytes become dysfunctional, leading to a reduction in insulin sensitivity and overall metabolic health [11,12]. In this context, understanding the mechanisms that govern adipocyte function and expandability is crucial for the development of new targeted therapies to improve insulin resistance and adipose metabolic health.

TRIB3 is a member of the Tribbles family of serine/threonine pseudokinases that functions as regulatory/scaffold proteins, controlling a plethora of metabolic and cellular functions (reviewed in [[13], [14], [15]]). Tribbles act as interaction platforms promoting and inhibiting post-translational modification, such as ubiquitination and phosphorylation, and affecting protein-protein interactions [16] by preventing or enhancing specific interactions. Thus, Tribbles have been shown to play a critical role in pathways that control cellular differentiation, lipid metabolism and immune cell activation among others [[17], [18], [19]]. Yet, the role of TRIB3 in adipocytes is incompletely understood. Previous *in vivo* studies have pointed to TRIB3 as a critical regulator of glucose tolerance and insulin sensitivity [[20], [21], [22], [23]], with inhibition of insulin-mediated AKT activation as the prime underlying mechanism in hepatocytes and adipocytes [[23], [24], [25], [26]], and in vitro studies have suggested a role for TRIB3 in adipocyte differentiation and function by regulating C/EBPβ [27] and PPARγ transcriptional activity [28]. Also of interest is the ability of TRIB3 to promote Wnt signalling by stabilizing the interaction between β-Catenin and TCF4 observed in colorectal cancer stem cells [29], as the Wnt pathway inhibits adipogenesis [10,30]. To determine the functional importance of TRIB3 in adipose tissue we therefore characterized the AT phenotype in a full-body TRIB3 knock-out mouse model and combined lipidomics, transcriptomics, interactomics and phosphoproteomics analyses to elucidate cell-intrinsic functions of TRIB3 in pre- and mature adipocytes. We found that TRIB3 ablation impairs adipocyte expandability and their lipid profile, resulting in an increased adipose tissue mass, composed of smaller adipocytes. In addition, we found that TRIB3 functions as an intermediate between molecules that regulate a number of critical signalling cascades in response to external stimuli. Our study presents TRIB3 as a critical signalling mediator that regulates AT expansion and homeostasis.

## 2 Materials and methods

### 2.1 Mouse experiments, licensing, husbandry and care

All experiments were performed in accordance with UK legislation under the Animals (Scientific Procedures) Act 1986. The University of Sheffield Project Review Committee approved all animal experiments which were carried out under the UK Home Office Project License 70/7992 held by Professor S.E. Francis, and Personal License to L. Martinez Campesino ID645D5F9. Mice were kept in an optimal and controlled environment to reduce stress. Mice were subjected to 12 h light/12 h dark cycle, at 22 °C with 40-60% of humidity. Their diet consisted of a standard chow (Harlan, 18% protein rodent diet) and it was unrestricted.

### 2.2 Mouse strains and genotyping

Development of full body *Trib3* knock out mice (*Trib3*^KO^) strain was previously described [31]. Genomic DNA isolated from mouse ear clips was amplified by PCR (Supplementary Table 1), using specific primers for the *Trib3* WT and KO alleles (Supplementary Table 2). Samples were qualitatively analyzed using a trans-illuminator with EtBr/UV filter (Supplementary Figure 1). All the genotyping process was performed by the Genomics Core facility. Mice were weighed and culled via pharmacological overdose of 0.2 ml sodium pentobarbital (200 mg/ml) applied into the peritoneal cavity and cervical dislocation. Body as well as tissue weights were recorded.

### 2.3 Magnetic resonance imaging

Fifteen-week-old chow-fed male *Trib3*^KO^ (n = 4) and *Trib3*^WT^ mice (n = 3) were subjected to magnetic resonance imaging (MRI). Images were obtained using a 9.4 T, Bruker Avance III MRI scanner (Bruker Biospin MRI GmbH, Ettlingen, Germany) with a 25 mm 1H volume coil. Each sacrificed mouse was placed in the center of the coil aligned with the abdomen and with the hips oriented at the top of the image. Structural MRI scans were performed using an MSME spin echo sequence (FOV 3.0 × 3.0 cm, 512x512 matrix, TE/TR 16 ms/1000 ms, Number of averages 16). A stack of contiguous axial slices of 1 mm thick were acquired for each mouse (35 ± 1 slices per mouse in total) and processed using Bruker Paravision 5.1 software. The slice package of MRI images was segmented and analyzed in FIJI/ImageJ. To align the fat measurements in the histograms, the location of the hips was used as standardized reference point. Intensity and threshold adjustment were performed for fat identification. Total adipose tissue, as well as subcutaneous (inguinal and dorsolumbar) and visceral (epididymal, mesenteric and perirenal) depots were distinguished based on their location.

### 2.4 Lipid profiling

Plasma was separated from isolated blood by centrifugation (1500×*g* for 5 min at room temperature) and immediately stored at -80 °C. For analysis, 150 μl of plasma was sent to the Department of Clinical Chemistry at the Royal Hallamshire Hospital (Sheffield Teaching Hospitals) to assess a full lipid profile measuring: total cholesterol, low (LDL) and high (HDL) density lipoproteins, triglycerides and glucose, using a Roche Cobas 8000 modular analyser series. For definitions of lipoprotein profiling see Supplementary table 3.

### 2.5 Glucose tolerance test (GTT) and insulin tolerance test (ITT)

Mice were fed on chow diet for at least 8 weeks and then fasted overnight. Fasting mice were weighted and blood was collected from the tail. A 20% glucose solution was prepared and filtered through 0.2 μm filter. For GTT, 2 mg of glucose were administrated per g of body weight and blood was collected at 0, 30, 60, 90 and 120 min after glucose challenge by tail sampling method. For ITT, mice were faster for at least 2 h and then weighted. Then 0.75 mU insulin per gram of body weight was injected intraperitoneally. Using an insulin syringe. Blood glucose was measured at 0, 20, 40, and 60, 90 and 120 min after injection.

### 2.6 Semi targeted lipidomics

3T3-L1 cells stably transfected with shScramble or shTRIB3 plasmids were differentiated in 6-well plates and once differentiated, cells were washed with 300 μL of ice-cold PBS solution three times. 500 μL of dry-ice cold methanol/water mix (80%/20%,v/v) was added and cells and scraped from the well. Cells were then collected and stored on dry ice or at -80 °C for lipidomic analysis.

### 2.7 Lipidomics

A volume of 50 μL homogenized cells was subjected to Liquid-Liquid extraction (LLE). The sample was vortex-mixed with methanol-methyl-tert-butylether (containing one internal standard per lipid class and an amount of antioxidant – BHT - to prevent lipid oxidation) after which an amount of water was added to induce phase separation. After incubation, the sample was centrifuged and the organic top layer containing all lipids was transferred to a clean sample vial. This lipid fraction was dried in a vacuum concentrator. Prior to analysis the lipid residue was dissolved in acetonitrile, thoroughly vortex mixed and transferred to an injection vial. LC-MS/MS sample analysis was conducted on an Ultimate 3000 UHPLC with LTQ-Orbitrap XL high resolution mass spectrometry detection. For chromatographic separation an Acquity BEH C18 column (2.1 × 100 mm, 1.7 μm) positioned in a 60 °C column oven was used. Upon injection of 5 μL sample a 10 min gradient was started (total runtime 20 min per sample). Sample analysis was conducted in both positive mode and negative mode. Generated data were submitted to MZMine for alignment and data analysis. Raw data was uploaded and normalized via Metaboanalist 5.0. Principal component analysis (PCA) analysis and heat map generation was also performed via Metaboanalist 5.0 software [32]. Enrichment analysis of normalized lipidomic data, using ‘ranking mode’, was performed via LIONweb [33]. Data has been deposited using Metabolights (www.ebi.ac.uk/metabolights/mtbls8891).

### 2.8 Isolation and culturing of primary mouse and human (pre)adipocytes

Adipose tissue from *Trib3*^KO^ and *Trib3*^WT^ was dissected, minced and digested using a collagenase buffer (HBBS medium (Gibco), 2% (v/v) BSA (Sigma) and 1.4 mg/ml collagenase type II (Sigma)) to facilitate the dissociation between adipocytes and stromal vascular cells (SVC). To separate and discard adipocytes from SVCs, the digested tissue was filtered and centrifuged, and the supernatant containing adipocytes was removed. The cell pellet corresponding to the SVC fraction was then incubated with red blood cell (RBC) lysis buffer, neutralized and centrifuged again. The resulting cell pellet containing SVCs was used for preadipocyte culture. The isolated SVC fraction from the AT digestion was cultured in complete DMEM media, changing media every 2 days. At 90% cell confluency, cells were treated with differentiation media, consisting of complete DMEM media supplemented with 1 μg/ml of Insulin (Sigma), 2.5 μM Dexamethasone (Sigma) and 0.5 μM 3-Isobutyl-1-methylxanthin (IBMX) (Sigma). After 4 days of differentiation, cells were maintained with complete media supplemented with 1 μg/ml of Insulin (Sigma) for 7 days, until complete differentiation.

Human liposuctions were isolated from subcutaneous adipose tissue of consented unrelated healthy volunteers aged between 20 and 50 years under ethical approval from the national Ministry for Human Resources in Hungary (Ref: FAT-H2020-001). Approximately 25 ml of fat was taken from liposuction material, centrifuged twice (430×*g*, 10min) in sterile PBS with the infranatant discarded. Enzymatic digestion was performed using collagenase (Sigma, 0.5 mg/ml in PBS) at 37 °C for 30-40 min with gentle rotation. The resultant homogeneous emulsion was filtered with a 100 μm strainer. To isolate adipocytes, warm culture media (DMEM with 10% FBS) was added to the filtered mixture for 5min to inhibit the enzymatic activity, then centrifuged (800×*g*), where the upper fat layer containing mature adipocytes was collected for further RNA isolation and gene expression analysis (RNeasy UCP kit, Qiagen). The remaining supernatant was discarded, and the cell pellet was collected for stromal vascular cell fraction (SVF) isolation. Red blood cell lysis was performed by resuspending the pellet with 10 ml of RBC lysis buffer (155 mM NH4Cl, 10 mM KHCO3, 0.1 M EDTA in H2O) for 10min. The cell pellet following centrifugation (800×*g*) containing the SVF was resuspended in sterile PBS + 2%(w/v) BSA. SVF was cultured to obtain a purified population of pre-adipocytes in DMEM with 10% FBS for 3-4 days at 37 °C and 5% CO2. Media was removed after 1 day of culture to discard non-adherent cells, then replaced twice weekly, until 80-90% confluent where cells were detached using trypsin/EDTA. Cell pellets were collected for further RNA isolation and gene expression analysis (RNeasy UCP kit, Qiagen).

### 2.9 RNA extraction and reverse transcription (RT)-qPCR

Total RNA was isolated from pre-adipocytes and mature adipocytes was performed by using the RNeasy lipid tissue mini kit (Qiagen) following manufacturer’s protocol. cDNA was synthesized using the Precision nanoScriptTM 2 RT kit (Primer design) according to manufacturer’s instructions. Quantitative PCR was carried out using Precision PLUS SYBR-Green master mix (Primer design) in a Bio-Rad i-Cycler machine. Specific primers were designed with NCBI BLAST and all assays were performed in triplicate and normalized to the expression levels of *cyclophilin A* as a suitable housekeeping gene. Fold changes compared to the house-keeping genes were calculated using ΔΔCt method. Amplification and melting curves were checked for each reaction to ensure specific single products were amplified with >90% efficiency.

### 2.10 RNA sequencing and bioinformatic analyses

RNA was isolated from differentiated adipocytes from *Trib3*^WT^ and *Trib3*^KO^ mice (N = 5, per group) using the RNeasy lipid tissue mini kit (Qiagen) according to manufacturer’s instructions. Samples were then sent to Novogene Co. Ltd (https://en.novogene.com) and used for messenger RNA sequencing. For cDNA library construction, mRNA was enriched using oligo(dT) beads, randomly fragmented following cDNA synthesis. Then cDNA libraries were assessed for quality control and qualified libraries were used for sequencing using Illumina sequencers (NovaSeq platform). The obtained raw data files were analyzed and quality controls on the fastq files, principal component analysis and differentially expressed gene analysis was performed. Data was analyzed using Ingenuity Pathway Analysis software (IPA, Qiagen). Data will be deposited in the NCBI GEO database and accession number will be provided upon submission of the manuscript.

### 2.11 Tissue sections and staining

Tissue sections were dewaxed in xylene, rehydrated in graded alcohols (100%–75% v/v) and rinsed in water following incubation in Gills hematoxylin solution for 5 min. The slides were then rinsed in running water, submerged in Scott’s Tap-Water for 30 s and rinsed in water again. Eosin-phloxine was used for counter-staining following water rinse and dehydration in graded alcohols and xylene. Slides were then mounted with coverslips using DPX mounting solution (Sigma-Aldrich, UK). Images to assess tissue morphology were taken using a brightfield microscope (Nikon Eclipse E6000) at 10x and 20x magnification. Three fields of view per tissue per mouse were captured and analyzed using Image J software. Adiposoft software [34] was used to asses adipocyte area.

### 2.12 Cell culture

The immortalized, murine-derived brown pre-adipocyte cell line (IBA) [35,36] was cultured in high-glucose (4.5 g/L d-glucose) DMEM medium (Life technologies, Carlsbad, CA) supplemented with 10% bovine serum and 1% penicillin and streptomycin. Cells were incubated in 5% CO_2_ incubator at 37 °C and 95% humidity. Generation of inducible TRIB3-tGFP IBA cells was done using third-generation lentiviral constructs using supernatants form HEK293T (ATCC CRL-3216, Manassas, VA, USA) cells transfected with lentiviral packaging plasmids. HEK293T cells were transfected using X-treme gene 9 DNA transfection reagent (Roche) following manufacturer’s instructions.

### 2.13 Western blot analysis

Western blotting was performed as described before [37]. In short, after induction with doxycycline, cells were lysed in iced-cold lysis buffer (150 mM NaCl, 1% NP40, 0.5% sodium DOC, 0.1% SDS, 25 mM Tris pH7.4 and supplemented with protease inhibitors). Protein concentrations were measured and samples were supplemented with Laemmli sample Buffer (LSB). Samples were run in SDS-PAGE gels and transfer to PVDF membranes. Blocking was performed in 5% milk in TBS-T for 45 min at room temperature. ECL western blot solution was used to detect protein expression using a LAS4000 Image Quant.

### 2.14 Immunoprecipitation for mass spectrometry

Immunoprecipitation was performed as described previously [38] using turboGFP-Trap beads (Chromotek) after 24/48 h induction with doxycycline. Samples were prepared in triplicates and Label Free Quantification (LFQ) was used to determine the interactors of TRIB3 and day 0 and day 6. Turbo-GFP only was used as control in both cases to filter out possible contaminants or proteins sticking to the beads or the tGFP tag.

### 2.15 Mass spectrometry

Mass spectrometry methodology was extensively described previously [38]. In short, precipitated proteins were digested with trypsin (250 ng/μL) and peptides were separated from beads using a C-18 stage tip (3 M, St Paul, MN, USA). After separation peptides were electro-sprayed directly into an Orbitrap Fusion Tribrid Mass Spectrometer (Thermo Scientific). The MS was run in DDA mode with one cycle per second. Full scan (400–1500 mass range) at a resolution of 240,000 Ions was performed reaching an intensity threshold of 10,000. Ions were isolated by the quadrupole and fragmented with an HCD collision energy of 30%. The obtained data was analyzed with MaxQuant [Version 1.6.3.4] using the Uniprot fasta file (UP000000589) of *Mus musculus* (Taxonomy ID: 10090).

### 2.16 Phosphoproteomics

Enrichment of phospho-peptides for SILAC labeling, IBA cells with inducible tFGP-TRIB3 overexpression were cultured in high-glucose (10% dialyzed FBS (BioWest)) DMEM (Thermo) lacking lysine and arginine supplemented with Lys-0/Arg-0 or Lys-8/Arg-10 (Silantes). Cells were lysed in 8 M Urea, 1 M Ammonium-BiCarbonate (ABC) containing 10 mM Tris(2-carboxyethyl)phosphine hydrochloride (TCEP) and 40 mM 2-chloro-acetamide supplemented with protease inhibitors (Roche, complete EDTA-free) and 1% (v/v) phosphatase inhibitor cocktails 2 and 3 (Sigma, Cat. No. P5726 and Cat. No. P0044). After ultra-sonication, Heavy and light cell lysates were mixed 1:1 and proteins (20 mg total) were over-night in solution digested with trypsin (1:50) (Worthington). Peptides were desalted using SepPack columns (Waters) and eluted in 80% acetonitrile (ACN). To enrich for phospho-peptides, 200 mg Calcium Titanium Oxide (CaTiO3) powder (Alfa Aesar, 325 mesh) was equilibrated 3 times with binding solution (6% Acetic acid in 50% ACN pH = 1 with HCl) after which the phospho-peptides were allowed to bind at 40 °C for 10 min on a shaker. After 6 times centrifugation and washing, phospho-peptides were eluted twice with 200 μl 5% NH3. The peptides were dried using a SpeedVac and the dissolved in buffer A (0.1% FA) before loading on in-house made C18 stage-tips and divided with high PH elution into three fractions (100 mM NH3/FA PH = 10 in 5%, 10% or 50% ACN).

### 2.17 Data analysis

Raw files were analyzed with the Maxquant software version 1.6.3.4 (Cox and Mann, 2008) with phosphorylation of serine, threonine and tyrosine as well as oxidation of methionine set as variable modifications, and carbamidomethylation of cysteine set as fixed modification. The Mus Musculus protein database of Uniprot (January 2019) was searched with both the peptide as well as the protein false discovery rate set to 1%. The SILAC quantification algorithm was used in combination with the ‘match between runs’ tool (option set at 2 min). Peptides were filtered for reverse hits and standard contaminants. Forward and Reverse ratios were plotted in R (www.r-project.org). The mass spectrometry proteomics data has been deposited to the ProteomeXchange Consortium via the PRIDE partner repository (http://www.ebi.ac.uk/pride, identifier: PXD046703).

### 2.18 F2 intercross mice study (BHF2) dataset analysis

Dataset generated at The university of California Los Angeles (UCLA) from the F2 intercross between C57BL/J6 and C3H/Hej mice (BHF2 population) on a ApoE insufficient background (ApoE^−/−^) is publicly available at http://www.genenetwork.org. Data was retrieved and Pearson correlations between male mice adipose tissue mRNA expression and the described phenotypes were obtained.

## 3 Results

### 3.1 *TRIB3* expression is increased in hypertrophic adipocytes and its expression correlates with different metabolic traits

First, we used publicly available human datasets from the Gene Expression Omnibus (GEO) repository to assess the expression of *TRIB3* in adipose tissue (AT). The expression of TRIB3 was evaluated as normalized signal intensity in three different datasets: GSE23699, GSE12050 and GSE61302. In GSE23699, the expression of genes in human subcutaneous adipose tissue was compared to the expression of human subcutaneous adipocytes. The study from GSE12050 evaluates the differential gene expression of tissue samples from lean and obese subjects. And GSE61302 contains microarray data from the analysis performed for the comparison of stromal stem cells before and after 7 days and 21 days of adipocyte differentiation in vitro.

**Figure 1:**
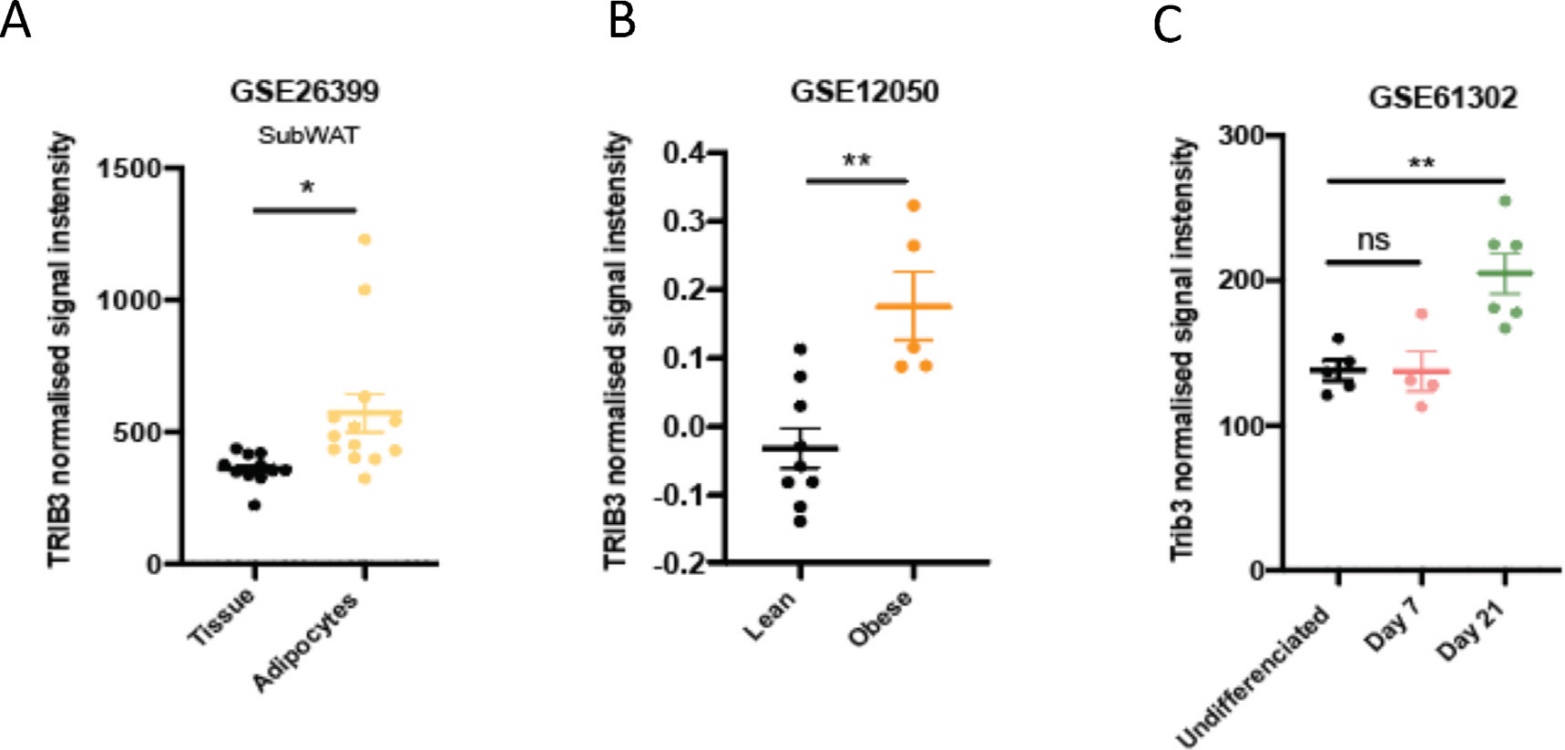
**Expression of TRIB3 in human adipose tissue and adipocytes.** (A) TRIB3 expression in human subcutaneous adipose tissue (GSE26399). (B) TRIB3 expression is higher in obese individuals compare to lean (GSE12050). (C) TRIB3 expression in ASCs (GSE61302). Graphs are presented as mean ± SEM, unpaired T-test (∗p ≤ 0.05. ∗∗p ≤ 0.01, N.S = not significant).

We found that TRIB3 is highly expressed in human AT, and in particular in the adipocyte fraction (Figure 1A). In addition, TRIB3 expression was elevated in AT of individuals with obesity compared to lean individuals (Figure 1B). Furthermore, TRIB3 expression was elevated in fully mature adipocytes (day 21) compared to undifferentiated adipose tissue-derived stromal stem cells (ASCs) and early differentiation state (day 7) (Figure 1C). In addition, we used a well-characterized mouse hybrid panel [39] to assess whether adipose tissue-specific TRIB3 expression correlates with any phenotypic trait [40]. We found that TRIB3 expression in AT positively correlates with leptin levels (r = 0.174; p = 4.1∗10^−2^) and negatively with weight (r = −0.170; p = 3.9∗10^−2^) and non-abdominal fat (r = −0.190; p = 2.1∗10^−2^) (Supplementary Figure 2); it should be noted that these traits are not independent (e.g. body weight positively correlates with leptin levels [41]). Altogether, these data point to TRIB3 as a potential regulator of AT function(s) and metabolic health in humans.

### 3.2 *Trib3* deficiency leads to an increased body weight, altered cholesterol and glucose homeostasis

**Figure 2:**
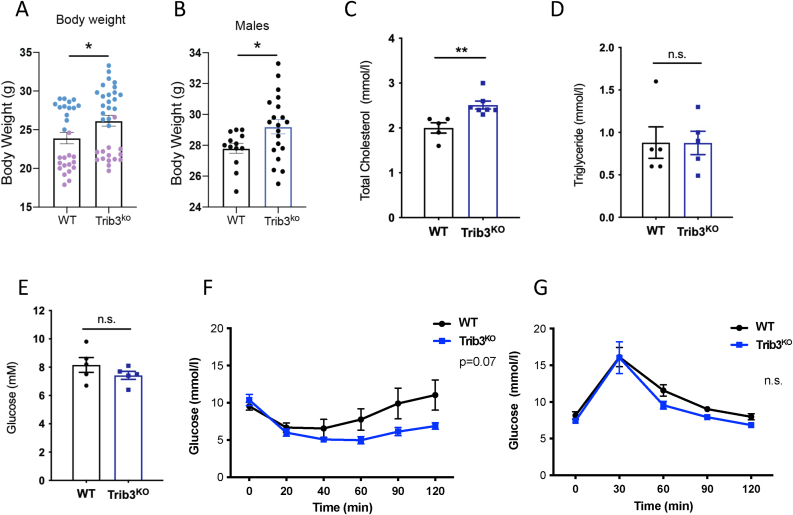
***Trib3***^**KO**^**mice show increased body weight and altered cholesterol and glucose homeostasis.** (A) Body weight of 15-week-old males and females from *Trib3*^KO^ and WT mice (n = 28–34). (B) Body weight of 15-week-old *Trib3*^KO^ and WT male mice (n = 13–20). (C) Cholesterol levels in plasma from *Trib3*^KO^ male mice compared to wildtype littermates (n = 5–7). (D) Plasma Triglyceride levels in *Trib3*^KO^ male mice compared to wildtype littermates (n = 5). (E) Fasting glucose levels in *Trib3*^KO^ male mice compared to wildtype littermates (n = 5). Graphs are presented as Mean ± SEM, unpaired T-test (∗p ≤ 0.05. ∗∗p ≤ 0.01. ∗∗∗p ≤ 0.001. ∗∗∗∗p ≤ 0.0001, N.S = not significant). (F) Insulin tolerance test. (G) Glucose tolerance test. Panels A-E: Unpaired T-test (∗p ≤ 0.05. ∗∗p ≤ 0.01. ∗∗∗p ≤ 0.001. ∗∗∗∗p ≤ 0.0001, N.S = not significant). For F and G, area under the curve (AUC) was calculated for each sample and an unpaired T-test was carried out to compare the impact of Trib3 deficiency on glucose handling.

While publicly available data show correlations between TRIB3 expression and adipocyte and AT function (Figure 1 and Supplementary Figure 2), such correlations may be indirect and either causal or consequential. Therefore, we investigated the role of TRIB3 in the AT more directly using *Trib3* full body knockout mice (*Trib3*^*KO*^*)*. Fifteen-week-old chow-fed *Trib3*^*KO*^ mice had an increased body weight (26.16 ± 0.71 g), compared to wild-type littermates (*Trib3*^*WT*^) (23.92 ± 0.74 g) (Figure 2A), in line with the negative correlation with body weight and the positive correlation with leptin levels observed in the mouse hybrid panel [39] (Supplementary Figure 2). However, when stratified by sex, only males had an increased body weight compared to wild-type littermates (Figure 2B), hence the focus on male mice for the rest of this study. *Trib3*^KO^ mice showed a significant increase in plasma total cholesterol (Figure 2C) but not in plasma triglycerides (Figure 2D) or fasting glucose levels (Figure 2E). When analyzing plasma lipoproteins, HDL levels were similar and LDL profiles showed some subtle yet significant differences, including LDL IVa, IIa and I (Supplementary Figure 3). In addition, glucose tolerance test (GTT) indicated no difference in glucose clearance in *Trib3*^KO^ mice compared to wild type animals (Figure 2F). Next, we performed an insulin tolerance test to assess insulin sensitivity in these animals and observed that *Trib3*^KO^ mice recover circulating glucose levels similar to the wild-type littermates after the insulin bolus (Figure 2G). Taken together, *Trib3*^KO^ mice display increased body weight and cholesterol levels that were not accompanied by altered plasma triglyceride levels or overt insulin resistance.

### 3.3 Adipose tissue mass and adipocyte size is affected in the *Trib3*^KO^ mice

**Figure 3:**
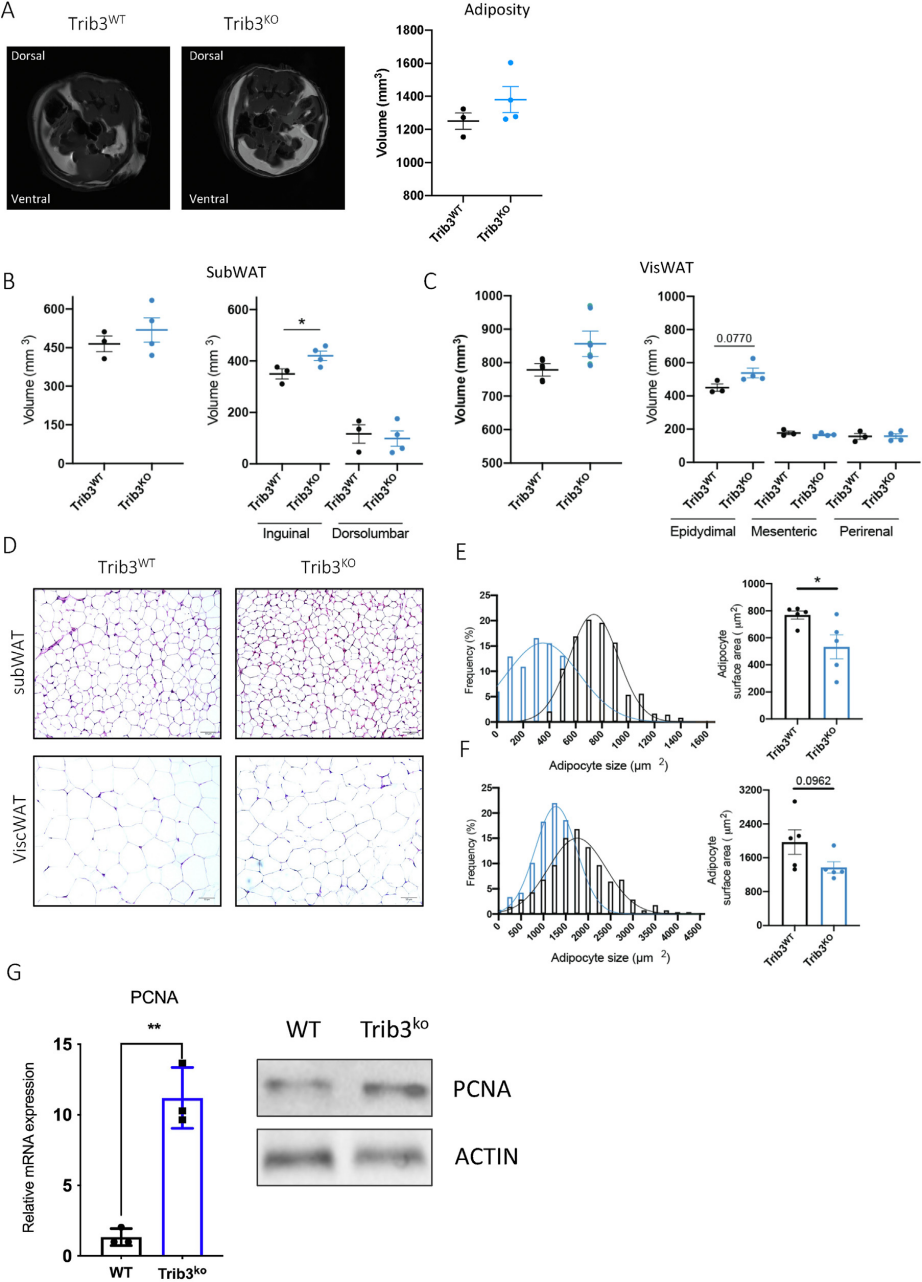
***Trib3***^**KO**^**mice display increased body weight and adiposity with smaller adipocytes.** (A) Representative MRI cross-sectional images showing the fat distribution at the abdominal region and total adipose quantification. (B) Quantitative analysis of the total volume and the individual depot volumes of the subcutaneous (B) and visceral (C) adipose tissue by MRI (n = 3–4). (D) Representative hematoxylin & eosin (H&E) stained subcutaneous (inguinal) and visceral (epidydimal) adipose tissue sections. Scale bar, 50 μm. Quantification of the mean adipocyte surface area and frequency distribution of adipocyte size of the H&E sections from the subcutaneous (E) and visceral (F) described depots (n = 5). Graphs are presented as Mean ± SEM, unpaired student’s T test, ∗p < 0.05. (G) PCNA expression and protein levels in subWAT.

To gain an insight into the potential anatomical reasons for the observed difference in body weights of male *Trib3*^*KO*^ *vs. Trib3*^*WT*^ mice, we performed magnetic resonance imaging (MRI) to assess body fat composition. Image slices were aligned at the hip level and the total adipose area per section was quantified, shown as white area within the image (Figure 3A and Supplementary Figure 4). The total fat volume per mouse showed a trend towards increased adiposity (Figure 3A), which was significant when fat depots were analyzed separately. In fact, we found that the difference in body weight is mainly due to an increase in the inguinal fat depot (subcutaneous white adipose tissue, subWAT; Figure 3B) and a moderate increase in epididymal WAT (visceral white adipose tissue, visWAT; Figure 3C). This difference in adiposity was also accompanied by a change in adipocyte size. Quantifications from H&E staining of sections from inguinal and epididymal fat depots (Figure 3D) confirmed that *Trib3* deficiency results in significantly smaller adipocytes in the subWAT with a shifted frequency distribution towards smaller cell size (Figure 3E). Similar differences, albeit less pronounced were found in visWAT (Figure 3F). Furthermore, the expression of Proliferating Cell Nuclear antigen (PCNA), a well-known marker of proliferating cells [42], was increased in the subWAT of *Trib3*^KO^ mice when compared to wild type littermates, both at the mRNA and protein levels (Figure 3G). Together these data suggest that TRIB3 plays a role in adipose tissue expansion and remodeling, with the strongest effects in subWAT where the absence of TRIB3 increases adipocyte proliferation and/or constrains cell size.

### 3.4 TRIB3 regulates the overall lipid profile of adipocytes

**Figure 4:**
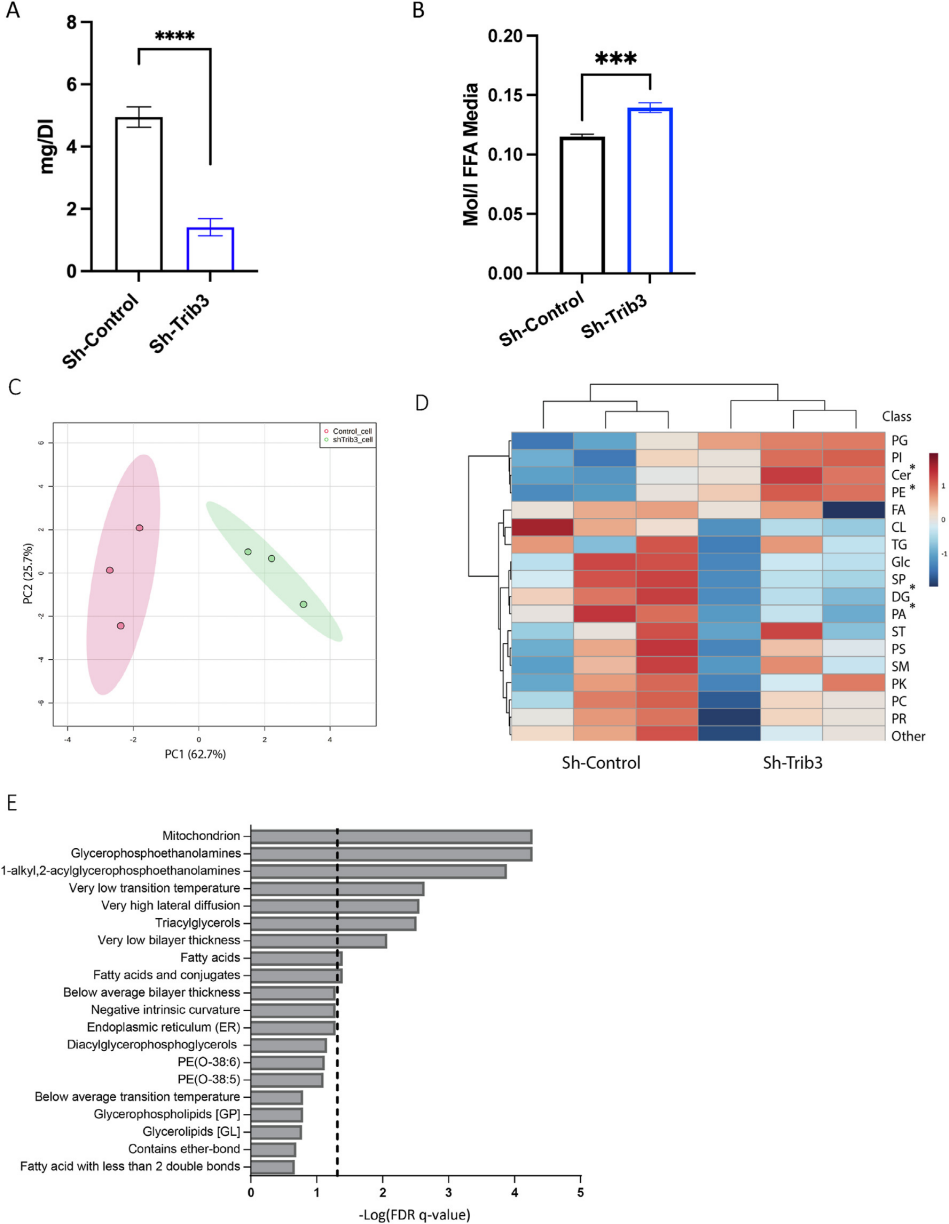
**Knockdown of *Trib3* in 3T3-L1 adipocytes alters lipid profiles.** (A). TG content of scrambled and *Trib3* shRNA KD 3T3-L1 adipocytes. (B) FFA levels in media of scrambled and *Trib3* shRNA KD 3T3-L1 adipocytes (C) Principal component analysis of Sh-*Trib3* and Sh-Control lipidome. (D) Semitargeted lipidomics analysis, identifying 577 lipid species total. Heat map of average lipid class abundance highlight *Trib3*’s total adipose lipid profile alteration. Statistical significance was calculated using unpaired T-test with Welch’s correction. (E) LIONweb enrichment analysis of semi-targeted lipidomic data ranking mode showing the top 20 enrichment hits, black line designating threshold for significance.

The reduced adipocyte size observed upon *in vivo Trib*3 ablation (Figure 3) could have different causes, including impaired expansion after cell division, an altered balance between lipid storage and release, or a combination thereof. To investigate these possibilities and exclude the influence of other cell types and/or organs we generated mouse 3T3-L1 (pre)adipocyte cell lines with stable shRNA-mediated knockdown (KD) of *Trib3* (Supplementary Figure 5). First, we measured the consequences of *Trib3* silencing on intracellular TG levels. Mature *Trib3* KD adipocytes displayed lower intracellular TG levels (Figure 4A) and higher levels of FFA in the media (Figure 4B), suggesting that *Trib3* is required to maintain a proper balance between lipid storage and release. To obtain a deeper understanding of the role of *Trib3* in lipid handling, we performed semi-targeted lipidomics, which allows in-depth total lipidome profiling by using lipid class standards to define the individual species within that class. *Trib3* KD in mature 3T3-L1 adipocytes resulted in changes in the lipid profile of these cells, as shown by initial principal component analysis (PCA), where the lipidome in KD cells clusters separately from control counterparts (Figure 4C). While intrinsic variability was observed for some lipid classes, the reduction of *Trib3* levels had consistent and significant effects on the levels of ceramides (Cer), phosphatidylethanolamines (PE), Glycosyldiradylglycerols (DG) and Glycerophosphates (PA) (Figure 4D and Supplementary Table 4). Both Cer and PE present membrane components, potentially pointing to altered membrane composition and thereby altered expandability. We further analyzed these data using LIONweb, an online ontology enrichment tool specifically designed to associate lipid species to biological features and functions [33]. Several processes and lipid classes linked to membrane functions were enriched (e.g. ‘Glycerophosphoethanolamines’ and ‘Very high lateral diffusion’ respectively); Figure 4E). Interestingly, the LIONweb category ‘Mitochondrion’ was most significantly enriched, suggesting a yet unidentified role of TRIB3 in cell biology (see Discussion). In summary, the reduction of *Trib3* levels alters the balance between lipid storage and release, and potentially also their expandability through altered lipid composition of membrane components, which together may present cell-autonomous mechanisms contributing to the smaller adipocyte size, observed in *Trib3*^KO^ mice (Figure 3).

### 3.5 Integrated omics identifies multiple TRIB3-dependent pathways regulating lipid handling and proliferation-differentiation in (pre)adipocytes

To identify cellular pathways that may mechanistically underpin the effects of *Trib3* silencing on the lipidome (Figure 4), ultimately resulting in altered adiposity and reduced adipocyte size *in vivo* (Figure 3), we employed a combination of omics approaches, including protein-protein interactome, phosphoproteome and transcriptome analyses.

**Figure 5:**
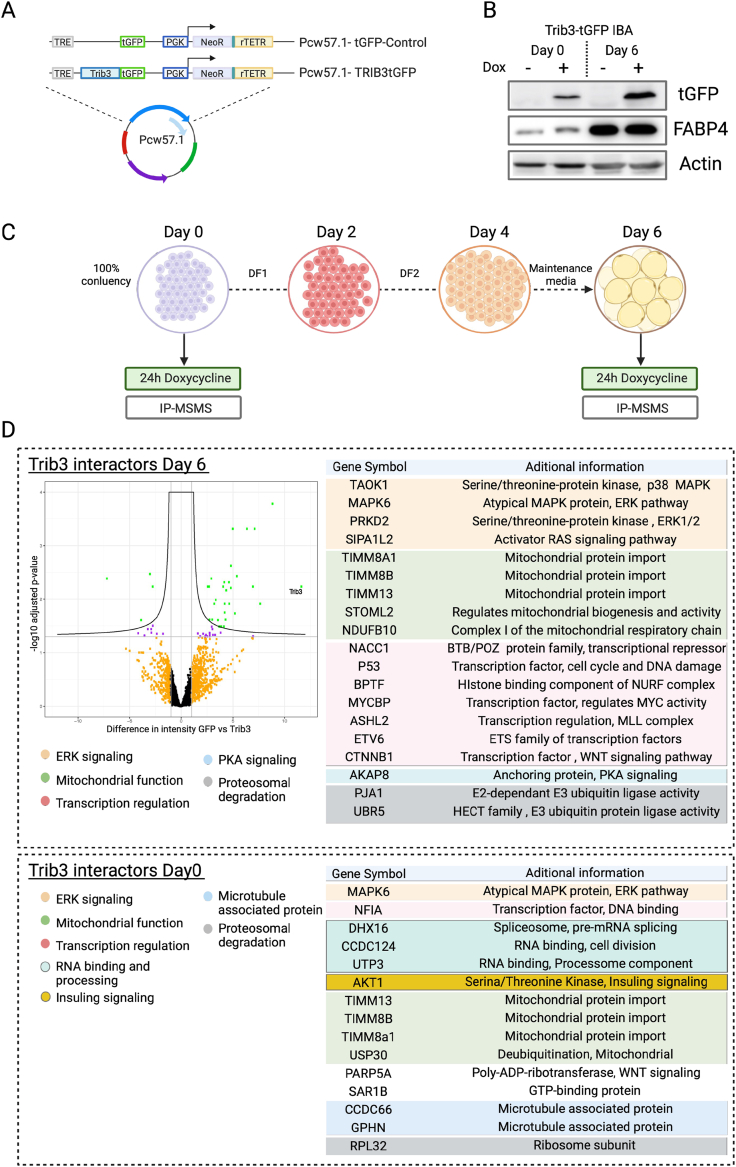
**Trib3 interacting proteins in (pre)adipocytes.** (A) Doxycycline inducible constructs used to generate TRIB3-tGFP and tGFP control cell lines in IBA cells. (B) Representative Western blots showing induction of TRIB3-tGFP upon doxycycline, together with FABP4 as an adipocyte differentiation marker and actin as loading control, at day 0 and day 6 of adipocyte differentiation. (C) Schematic representation of adipocyte differentiation. In short, cells were cultured until 100% confluency was reached then cells were treated with differentiation media DF1 (1 μg/ml of Insulin, 2.5 μM Dexamethasone and 0.5 μM IBMX). After 2 days, cells were treated with DF2 (complete media supplemented with 1 μg/ml of Insulin) for 2 days and finally at day 4 cells were kept in maintenance media (DMEM high glucose, 10% FBS). (D) Table of TRIB3 interacting proteins in undifferentiated (Day 0) and differentiated (Day 6) IBA (pre)adipocytes. Additional interactome data are provided as Supplementary file 1.

We first analyzed the TRIB3 interactome, employing our previously described mass spectrometry-based approach [38]. We generated inducible TRIB3-tGFP cell lines in mouse IBA (pre)adipocytes, an accessible and versatile model to study adipocyte biology [36], and induced TRIB3-tGFP expression with doxycycline for 24 h at day 0 and day 6 of adipocyte differentiation (Figure 5A); differentiation was monitored by expression of Fabp4 (Figure 5B). Subsequently, we identified TRIB3 interacting proteins by immunoprecipitating TRIB3-tGFP with a nanobody against tGFP, coupled to agarose beads, followed by mass spectrometry analysis of the immunoprecipitated proteins (Figure 5D and Supplementary file 1). Among the interactors found at day 0, two serine/threonine protein kinases are of particular interest: the Serine/Threonine kinase 1 (AKT1), a previously reported TRIB3 interacting protein that regulates –amongst others– insulin signalling [26], and MAPK6/ERK3, an atypical MAP kinase that is member of the extracellular-regulated kinases. Other interacting proteins included a number of proteins involved in RNA binding and processing (DHX16, CCDC124 and UTP3) and microtubule associated proteins such as CCDC66 and GPHN. Interestingly, we also detected mitochondrial importer proteins of the TIMM/TOMM complex that we have previously identified as TRIB3 interacting proteins in cancer cells [38] and USP30, a deubiquitylation enzyme involved in mitochondrial fusion [43]. Similar to the interacting proteins identified prior to differentiation, we found that TRIB3 interacts with ERK/MAPK pathway proteins in mature adipocytes, such as TAOK1, MAPK6, PRKD2 and SIPA1L2, confirming the central regulatory role of TRIB3 on ERK/MAPK signalling described by us and others in multiple cellular contexts [[44], [45], [46], [47]]. In addition, we found the same mitochondrial transporters (TIMM13, TIMM8A1 and TIMM8B), together with two other mitochondrial proteins (STOML2 and NDUFB10) interacting with TRIB3 in mature adipocytes. In contrast with the interacting proteins detected at day 0, at day 6 we found a high number of proteins that have been linked to transcriptional regulation such as NACC1, ASHL2, ETV6 and CTNNB1/β-catenin. NACC1 and ETV6 are associated with transcriptional repression and have been described in ovarian cancer progression [48,49] and leukemia [50] respectively, neither of these proteins have been reported as an interactor of TRIB3 before. In contrast, ASHL2 and CTNNB1/β-catenin have been previously described as TRIB3 interacting partners. ASHL2 is a member of the WRAD complex, responsible for histone-3 lysine-4 methylation in mammalian cells, and we and others have previously reported the interaction between TRIB3 and subunits of the MLL-WRAD complex [38,51,52]. Previous studies have also reported the interaction between TRIB3 and β-catenin, showing that TRIB3 promotes Wnt signalling by interacting with CTNNB1/β-catenin and TCF4 and enhances their transcriptional activity [29,53,54]. In adipocytes, Wnt signalling regulates the balance between proliferation and differentiation, promoting pre-adipocyte proliferation whilst inhibiting terminal differentiation into mature adipocytes [10,30]. Taken together, the (pre)adipocyte TRIB3 interactome spans many different protein classes (e.g. kinases and transcriptional regulators) as well as cellular localizations (e.g. cytoplasm, nucleus and mitochondria), which may directly or indirectly link to proliferation, cell size and lipid storage and release.

**Figure 6:**
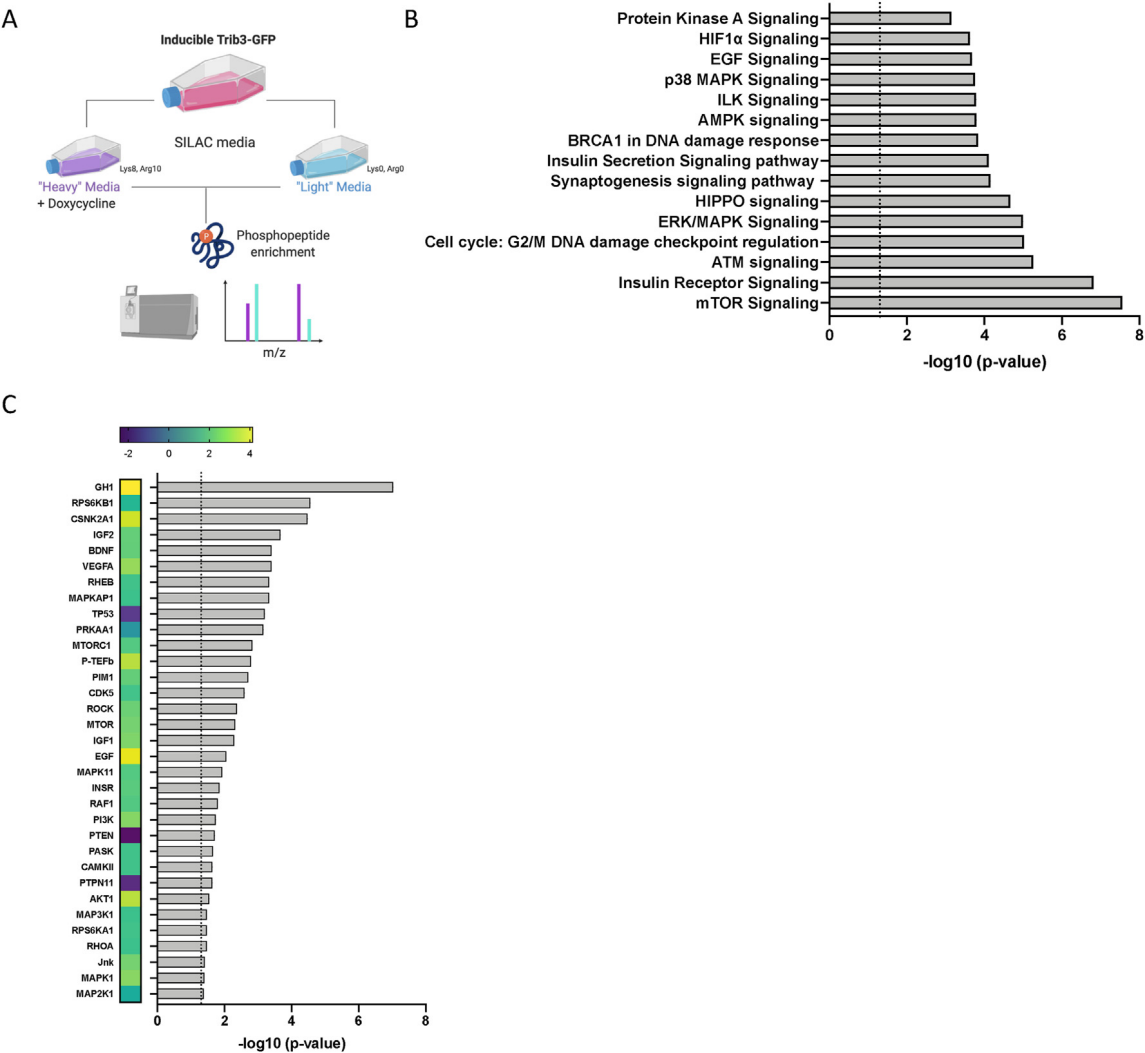
**TRIB3 induction alters mTOR and MAPK signalling and affects adipocyte cell cycle progression.** (A) Schematic representation of phospho-proteomics experiments using TRIB3-tGFP IBA cells in heavy and light SILAC media. (B) Top 15 altered canonical pathways found dysregulated upon TRIB3 induction in phospho-proteomics experiment in IBA cells. The dotted line indicates a p-value of 0.05. (C) Altered upstream kinases, protein complexes and enzymes according to Ingenuity pathway analysis (Quiagen) indicating p-value (dotted line indicates p-value of 0.05) and activation Z-score (colour coded). (For interpretation of the references to colour in this figure legend, the reader is referred to the Web version of this article.)

Since interactome analyses may be limited by the strength and stability of protein-protein interactions, we complemented the TRIB3 interactome studies with TRIB3-dependent phosphoproteome analyses. While TRIB3 is not able to phosphorylate target proteins due to the lack of the metal binding motif in the kinase domain, it can interact with canonical kinases and regulate their function [47,55], as also observed here (Figure 5). We again used the inducible TRIB3-tGFP IBA cells described above and subjected these to SILAC-based quantitative proteomics. Cells were maintained in media containing heavy or light amino acids (lysine and arginine) for 5 passages and incorporation of labelled amino acids was assessed prior to the beginning of the experiment (data not shown). TRIB3-tGFP lines were induced with doxycycline for 24 h in differentiated IBA cells, and then the phospho-proteome of induced vs uninduced differentiated TRIB3-tGFP cells was compared, together with the reverse experiment (Figure 6A). Among the top 15 canonical pathways that were identified, dysregulated mTOR and insulin receptor signaling were the most significant (Figure 6B and Supplementary file 2), in line with the interactome data presented above (Figure 5) and previous studies [26,56]. In addition, cellular pathways that control whole-body energy balance were found to be dysregulated, including the ERK/MAPK, AMPK and PKA pathways. AMPK (AMP-activated protein kinase) inhibits fatty acid and cholesterol synthesis in adipocytes upon low levels of nutrients [57]. On the other hand, PKA is a major regulator of mitochondrial biogenesis and lipolysis, enhancing browning of WAT and the release of fatty acids from the lipid droplets by phosphorylation of lipases and perilipin [58,59]. Moreover, the induction of TRIB3 affects the G2/M DNA damage checkpoint and ATM signaling, which, together with the result from the previous section where p53 was found as an interacting partner of TRIB3, situates the pseudokinase as a pivotal regulator of the cell cycle in adipocytes. Analysis of the upstream regulators reveals kinases and other protein complexes that are altered by TRIB3 in adipocytes (Figure 6C). Among the most significant upregulated are EGF, AKT1, GH1 and MAPK1, it is also worth mentioning the downregulation of p53, PTEN and PTPN11 according to the Z-score generated. We also found several transcription factors that have changed their phosphorylation status upon TRIB3 induction, including ATF4, ATF7 and STAT3. These results indicate that TRIB3 is able to regulate adipocyte function, modulating the activation of mTOR, insulin signalling and ERK/MAPK pathways, resulting in changes in transcription factor activation that drive adipocyte function.

**Figure 7:**
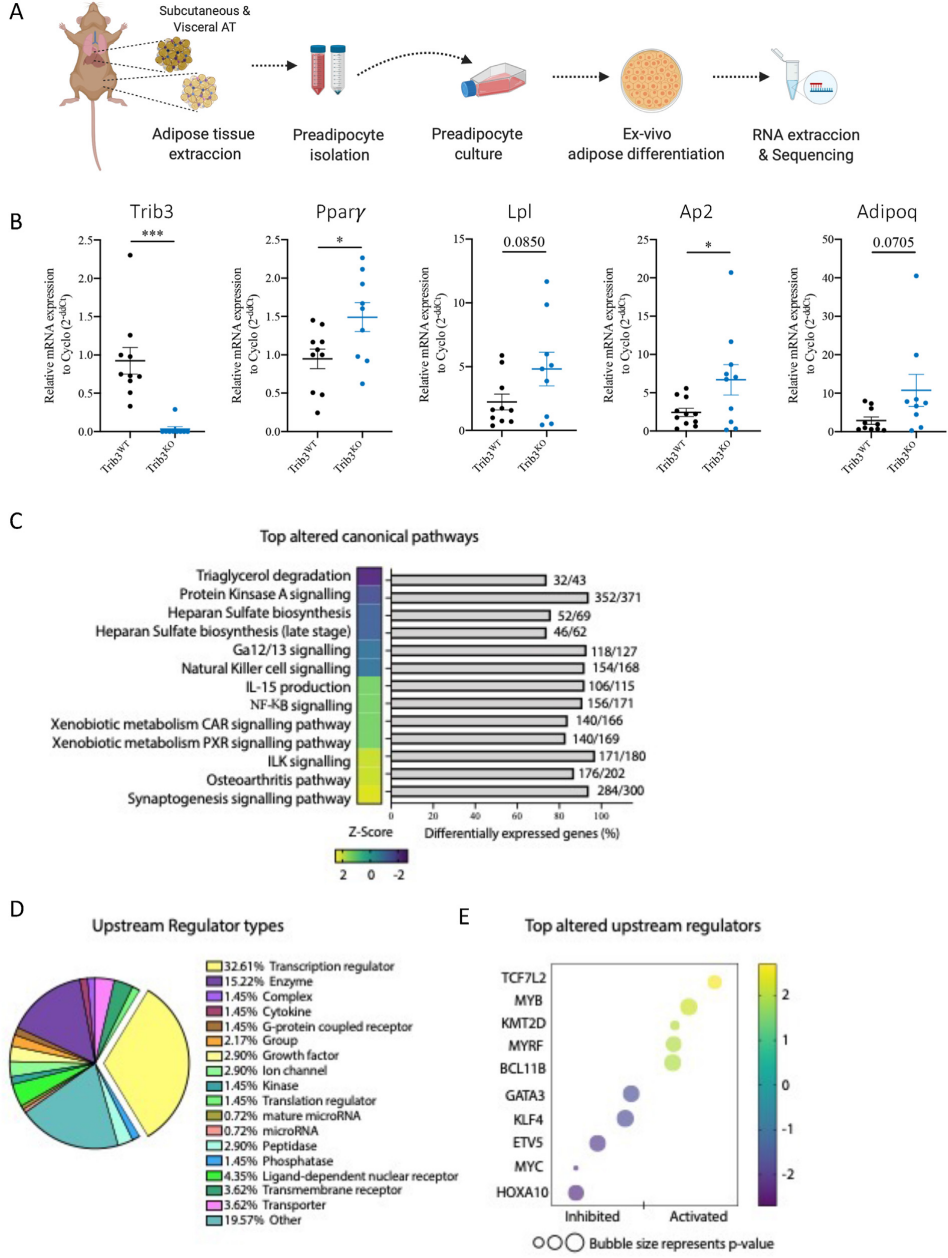
**RNA sequencing of *ex-vivo* differentiated adipocytes reveals differences in gene expression profiles between *Trib3***^**KO**^**adipocytes and wild type.** (A) Schematic representation of preadipocyte isolation and ex-vivo differentiation. (B) Expression of *Trib3*, *Pparγ, Lpl, Fabp4* and *Adipoq* in ex-vivo differentiated adipocytes. Cyclophilin A expression was used as an internal control. Graphs are presented as mean ± SEM, unpaired student’s test (∗p ≤ 0.05. ∗∗p ≤ 0.01. ∗∗∗p ≤ 0.001. ∗∗∗∗p ≤ 0.0001, N.S = not significant) (C) Top altered canonical pathways found dysregulated in the RNAseq of the ex-vivo differentiated subWAT of the Trib3^ko^ mice compared to WT littermates. (D) Upstream regulators analysis of RNA-seq dataset using Ingenuity pathway analysis (Qiagen). (E) Top altered upstream regulators indicating p-value and activation Z-score.

Finally, we analyzed the effect of *Trib3* depletion on adipocyte gene expression. To focus on cell-intrinsic effects rather than changes caused by interplay of adipocytes with other AT-resident cells, the stromal vascular fraction (SVF) from subcutaneous and visceral WAT depots from *Trib3*^*KO*^ and *Trib3*^*WT*^ male mice was isolated, expanded and differentiated into mature adipocytes as shown before [60](Figure 7A). Adipocyte differentiation was evaluated by mRNA expression of markers such as *Pparγ, Lpl*, *Fabp4 and Adipoq* (encoding adiponectin). We found that *Trib3* deficient cells displayed significantly higher levels of *Ppa*r*γ* and *Fabp*4 when compared to WT cells, while *Adipoq* and *Lpl* showed a strong but not significant trend of upregulation in the *Trib*3 deficient cells (Figure 7B). When the same markers were assessed in *ex-vivo* differentiated SVF from visceral WAT similar trends were observed albeit to a lesser extent (Supplementary Figure 6), in line with the stronger effect of *Trib3* ablation on subcutaneous AT volume and adipocyte size compared to vWAT (Figure 3B–F). We also analyzed correlations between TRIB3 mRNA expression and various markers in *ex vivo* differentiated human adipocytes, starting from the SVF of subcutaneous AT. Similar trends were observed to mouse AT, which did not reach statistical significance due to the low sample size or larger individual variation in humans compared to mice (Supplementary Figure 7).

To gain a more comprehensive insight into *Trib3*-dependent changes in adipocyte transcriptional programmes, transcriptome analyses of *ex vivo* differentiated adipocytes from subcutaneous WAT of *Trib3*^*KO*^ and *Trib3*^*WT*^ mice were performed. Pathway analysis of differentially expressed genes (Ingenuity Pathway Analysis (Qiagen)) was performed and revealed 41 altered canonical pathways and 64 diseases and functions significantly affected by *Trib3* deficiency (Supplementary Table 5). The top altered canonical pathways in *Trib3*^*KO*^ adipocytes include downregulated pathways involved in lipid homeostasis (TG degradation and PKA signaling) in line with our lipidome, interactome and phosphoproteome data (Figure 4, Figure 5, Figure 6). Among all the molecules that were found significantly dysregulated (p-value <0.05) ‘transcription regulator’ was the most frequent molecule type identified (Figure 7D). Within this category, the top activated molecule was TCF7L2 while HOXA10 was the most inhibited (Figure 7E). TCF7L2 (also known as TFC4) is the endpoint of the Wnt/β-Catenin signalling cascade which has been implicated in adipocyte differentiation and function [61,62] as well as the Wnt/β-Catenin pathway being associated with TRIB3 in multiple other cell types [29,53,54]. The homeobox transcription factor HOXA10 has also been implicated in adipocyte differentiation and function [63].

Taken together, our integrated omics approaches indicate that i) TRIB3 can interact with a large set of proteins in adipocytes, including various kinases, such as AKT and MAPK6, and transcriptional regulators, such as CTNNB1//β-catenin, ii) TRIB3 can directly or indirectly alter signaling pathways in adipocytes, such as mTOR, MAPK and PKA signaling, iii) depletion of *Trib3* affects the adipocyte transcriptome, with dominant effects on the transcription factors TCF7L2 and HOXA10. Rather than pointing to a single cellular pathway being affected, these findings support TRIB3 playing multiple roles, both in the cytoplasm and the nucleus and potentially also in mitochondria, in intertwined pathways, ultimately contributing to an optimal balance in proliferation vs. differentiation capacity, and proper lipid storage. As a consequence, ablation of *Trib3* affects the proliferation-differentiation balance and net lipid storage in the AT of *Trib3* full body knockout mice.

## 4 Discussion

*In vivo* studies in humans and mice have indicated that the pseudokinase TRIB3 regulates energy metabolism [64,65], and cell-based studies suggest a role in adipocytes [28]. However, it was unknown if these observations are causally linked. In our study, we investigated the impact of whole-body ablation of Trib3 in mice and found that it leads to adipose tissue expansion while maintaining insulin sensitivity. The KO animals exhibited an increase in the number of smaller adipocytes, a phenotype often associated with insulin sensitivity [[66], [67], [68]]. Through a combination of lipidome, interactome, phosphoproteome and transcriptome analyses, we uncovered a multifaceted role for TRIB3 in proliferation, TG storage and cellular expansion. These functions are potentially regulated through multiple cellular pathways, including MAPK/ERK and PKA signalling and TCF7L2/beta catenin-mediated gene expression. In support of these various molecular roles, TRIB3 has been reported to be localized in the cytoplasm and the nucleus [44], and to interact with a large set of cellular proteins in various biological settings [29,69]. Interestingly, the TRIB3 interactome in adipocytes reported here as well as the interactomes reported earlier in MCF7 breast cancer cells and HEK297T cells [38], all suggest a potential role for TRIB3 in mitochondria, which will be the focus of future studies.

Our combined interactome and phosphoproteome analyses indicate that TRIB3 functions as an integrator of signalling pathways in adipocytes, capable of regulating kinases downstream of membrane receptors, such as AKT1, mTOR or ERK3, as well as transcription factors such as CTNNB1 or ASH2L that drive transcription of key factors of adipose biology. Regarding the interactions with kinases and proteins that regulate kinases, TRIB3 appears to influence their substrate specificity, redirecting these kinases in certain directions [70,71]. The effect of TRIB3 on transcription factor activity is also complex. TRIB3 has been shown to interact and modulate the activity of a number of transcription factors [29,72], and can repress transcription through recruitment of repressor proteins like ZBTB1 [38] or interference with recruitment of transcriptional activators like the MLL complex [73]. In agreement with other studies [29] we describe an interaction between TRIB3 and CTNNB1/β-catenin (Figure 5) and also report upregulation of TCF7L2-mediated transcription upon ablation of *Trib3* (Figure 7E). Together, these findings suggest that TRIB3 may negatively regulate Wnt signalling in adipocytes, potentially recruiting aforementioned repressor proteins or preventing CTNNB1//β-catenin from entering the nucleus. However, further experimental studies are necessary to fully understand this model, particularly since Wnt activity impairs adipogenesis [10,30] and TRIB3 has been described as a positive regulator of Wnt signalling in various cancer types [29,53,54].

Smaller adipocytes, as observed here in *Trib3*^KO^ mice, have been associated with insulin sensitivity [[66], [67], [68]]. In fact, various therapeutic approaches have aimed to increase adipocyte proliferation to generate more small adipocytes [74]. While this suggests that targeting TRIB3 in adipocytes for degradation may represent a rational therapeutic approach; it should be noted that drug targeting of Tribbles and specifically TRIB3 presents both an exciting area of development and a significant challenge, as discussed elsewhere [15,[75], [76], [77]]. Furthermore, it is important to consider that adipocyte-specific targeting of TRIB3 may be required and the role of TRIB3 in AT resident immune cells, which can also affect adipocyte size and functionality [78], has not been addressed directly yet. While the current study supports the view that TRIB3 is a critical regulator of adipocyte proliferation, homeostasis and function, future studies are clearly needed to elucidate its therapeutic potential.

## Author contribution statement

MHQ, LMP, IM, ZI, SR, PSA, ECAS, RE and HV performed the experiments, data-analysis, and wrote the manuscript; AV and JV performed patient inclusion and detailed clinical assessment. NST, HLW, EKT and EK designed and supervised the study. All authors reviewed the manuscript.

## Declaration of competing interest

The authors declare that they have no known competing financial interests or personal relationships that could have appeared to influence the work reported in this paper.

## Data Availability

Data will be publicly available upoin acceptance of manuscript.
