# Supplementary material for: The pseudokinase TRIB3 controls adipocyte lipid homeostasis and proliferation in vitro and in vivo
Source: Mol Metab. 2023 Oct 30;78:101829. doi: 10.1016/j.molmet.2023.101829 (PMC10663684; doi:10.1016/j.molmet.2023.101829)
Supplement: Multimedia component 1 [file mmc1.pdf]

Supplementary material

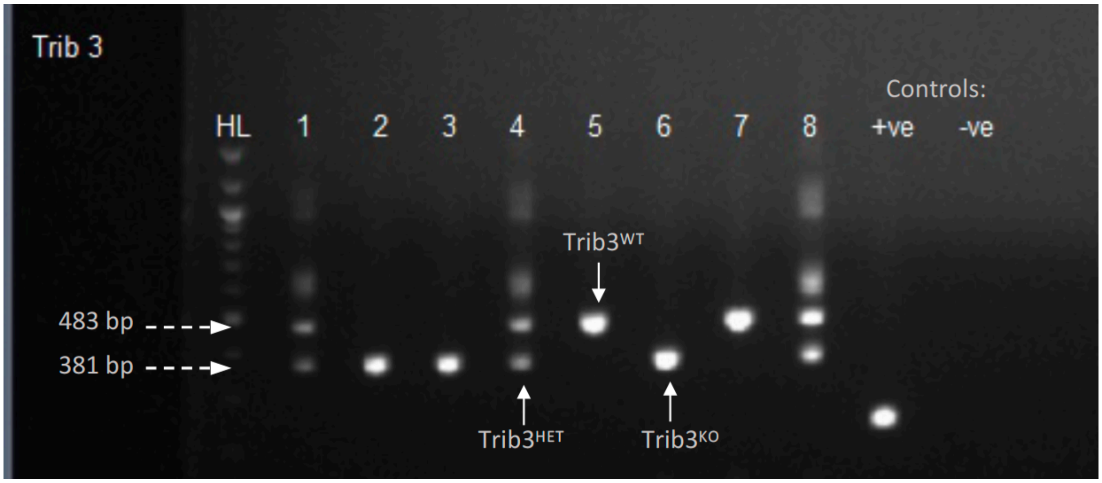

Supplementary Figure 1 Trib3 genotyping results. Result example to assess the genotype of mice from the *Trib3*KO strain. As it is shown, a single band at 381bp corresponds to the mutant allele (*Trib3* with the gene-trap vector inserted – *Trib3*KO), while the band at 483bp corresponds to the *Trib3*WT allele. The presence of both bands indicates heterozygosis (*Trib3*HET). The gel also includes positive and negative controls to ensure the reliability of the PCR.

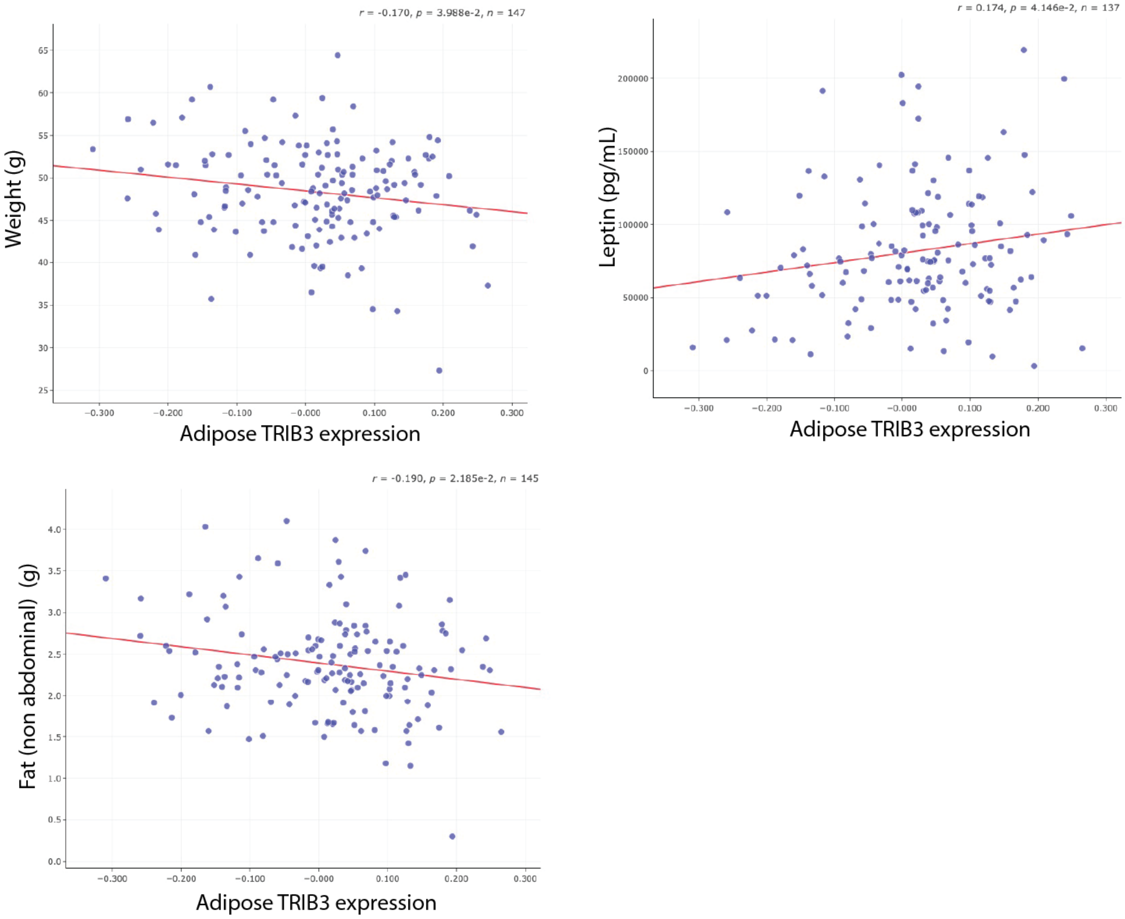

Supplementary Figure 2 Correlation between TRIB3 expression and phenotypic traits. Pearson correlations between TRIB3 expression in mouse white adipose tissue (WAT) and body weight, leptin levels and non-abdominal fat from an F2 intercross study (BHF2 population).

A

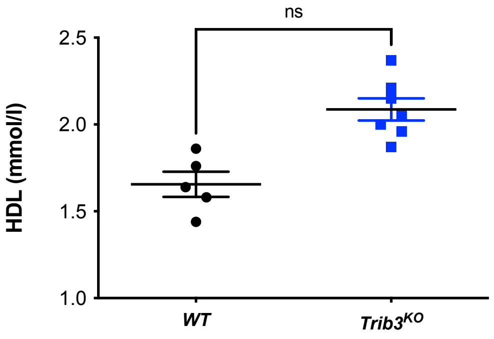

B

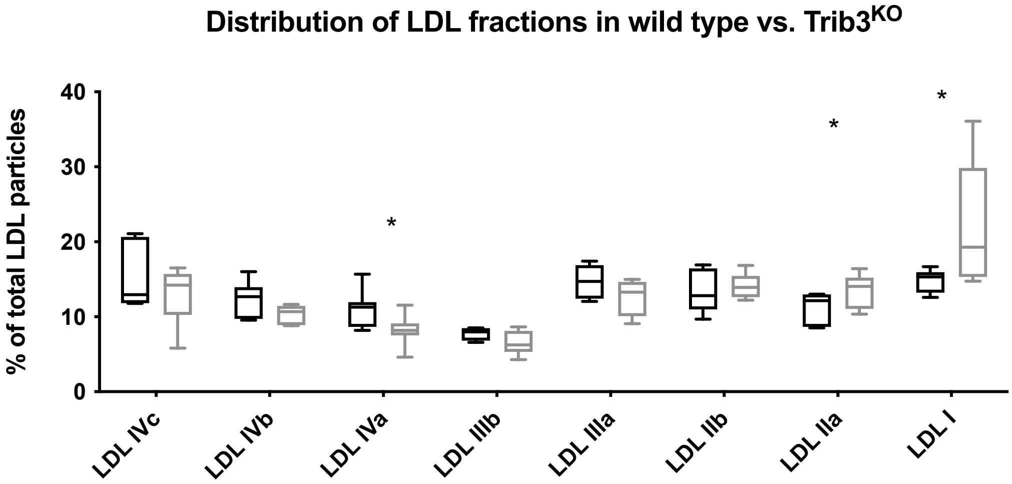

Supplementary Figure 3 Lipoprotine profile in *Trib3*^{KO} vs. wild type animals. (A) HDL levels. One way ANOVA with selected pairs testing. (B) Comparison of the distribution of LDL fractions in wild type (black bars) vs. *Trib3*^{KO} (blue bars) (N=7) mice.

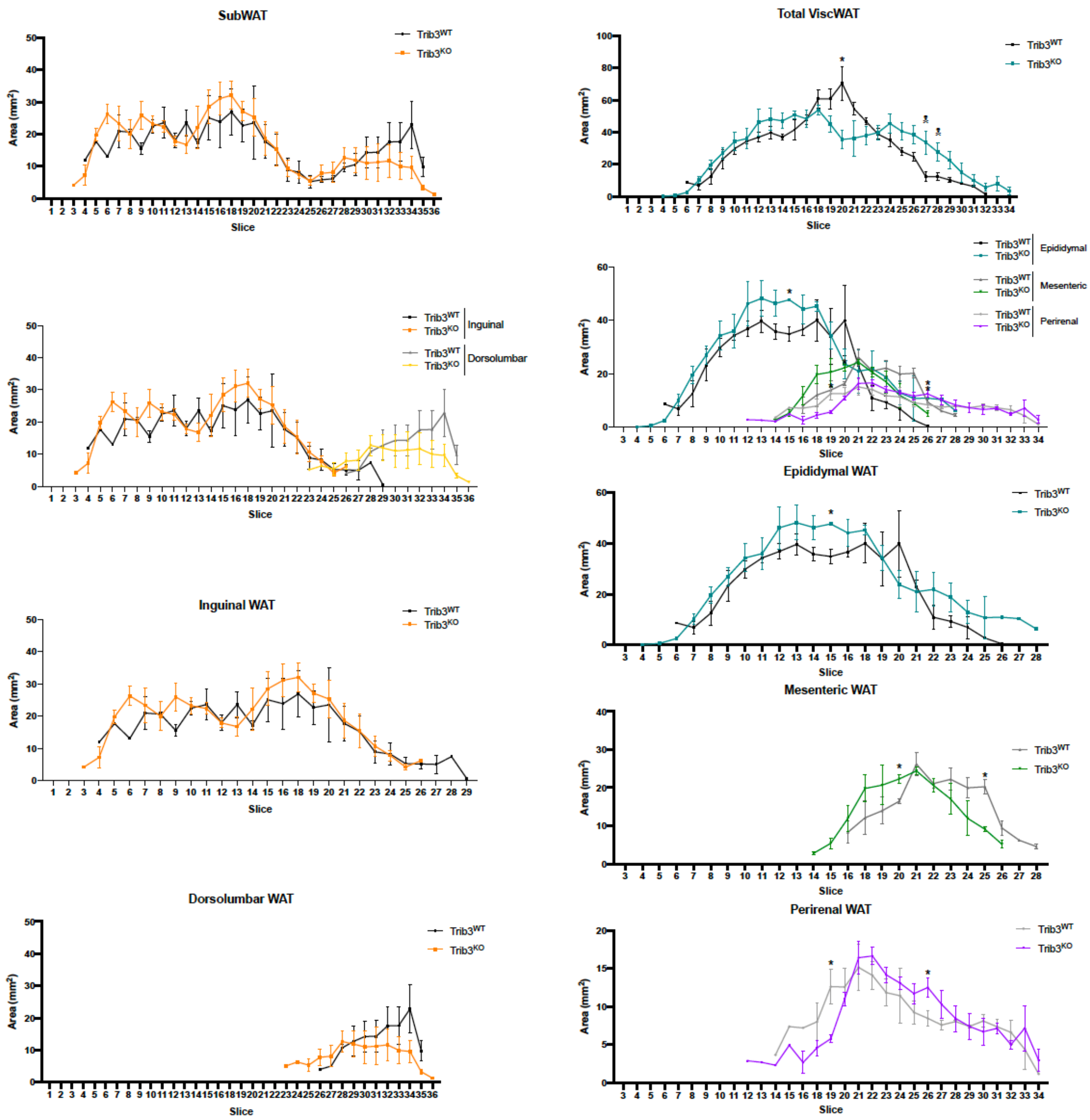

Supplementary Figure 4 MRI analysis of individual AT depots. Quantification of adipose area per slice within the different coronal regions, distinguishing between Subcutaneous (left, inguinal + dorsolumbar) and visceral (right, epididymal + mesenteric + perirenal) depots. Graphs are presented as mean ± SEM, unpaired student's T test, *p<0.05.

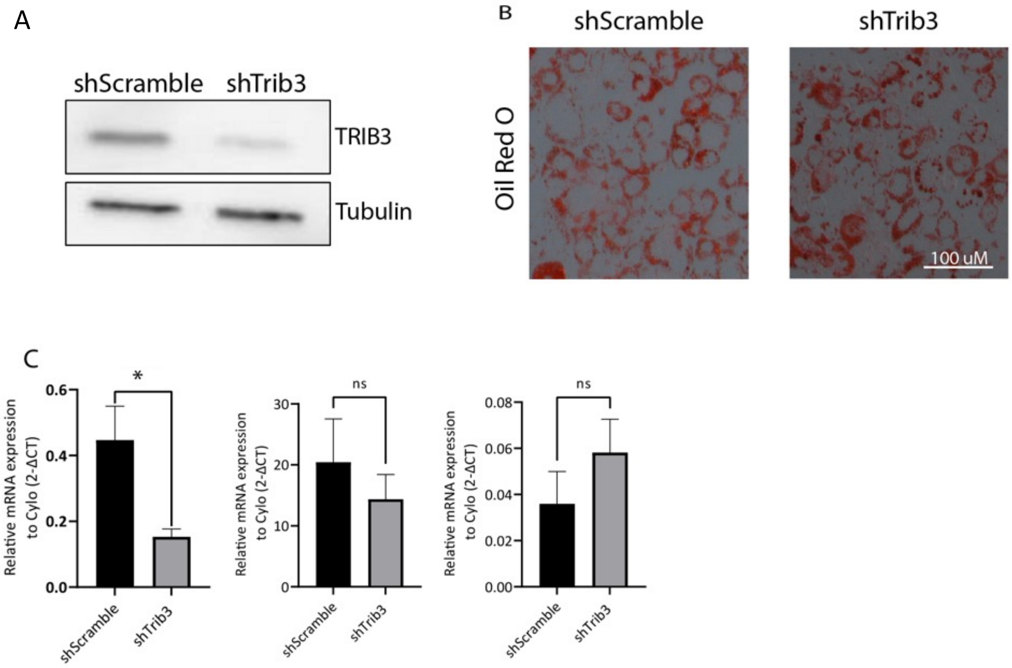

Supplementary Figure 5 Characterization of Trib3 KD in mature 3T3-L1 adipocytes. (A) Trib3 protein levels in 3T3-L1 cells scramble control and sh-Trib3 cells. (B) Oil red O staining of sh-Scramble control and sh-Trib3 cells. (C) mRNA expression of Trib3, Fabp4 and Pparg in sh-Scramble and sh-Trib3 3T3-L1 cells. Graphs are presented as mean ± SEM, paired student's T test, *p<0.05.

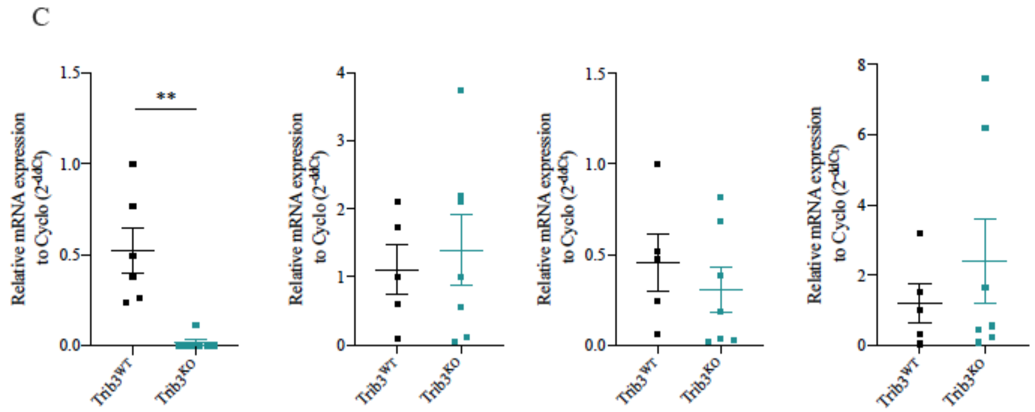

Supplementary Figure 6 Expression levels of mouse *Trib3, Pparg, Lpl* and *Fabp4*. Analysis of mRNA was performed in ex-vivo differentiated WAT from WT and Trib3^{KO} mice. Graphs are presented as mean ± SEM, unpaired student's T test, **p<0.001.

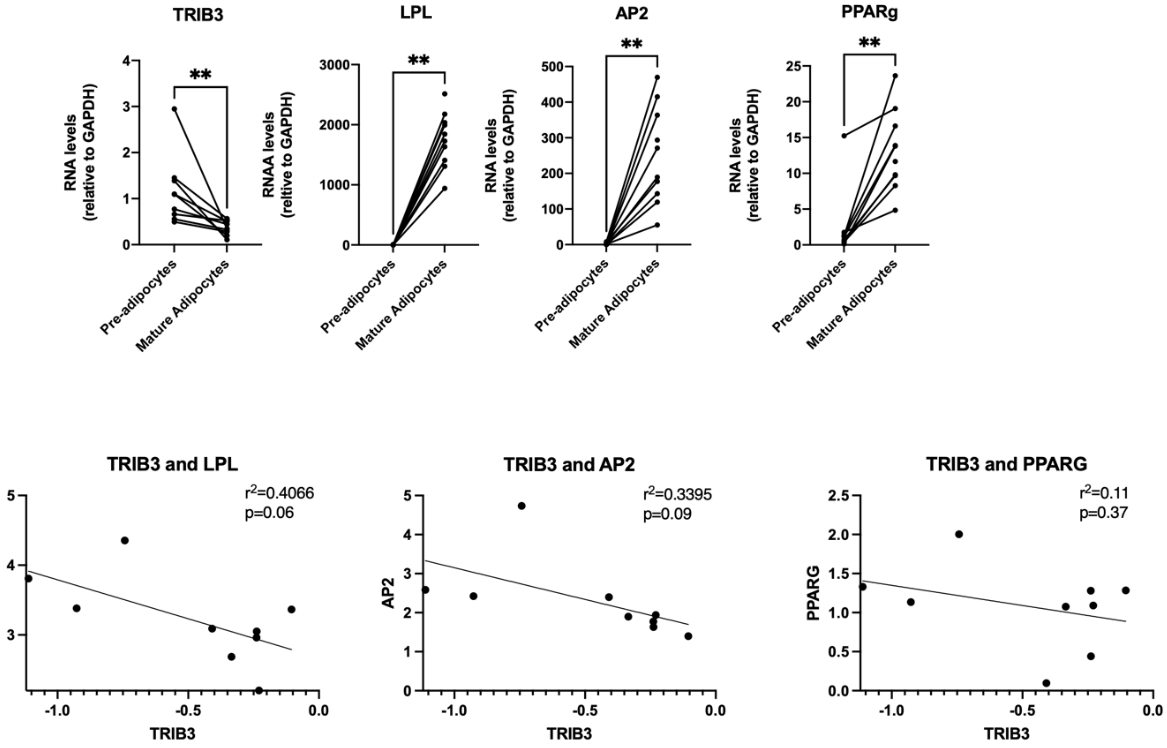

Supplementary Figure 7: Expression levels of human *TRIB3, PPARG, LPL* and *AP2/FABP44*

(A) Analysis of mRNA expression in ex-vivo differentiated WAT from human SVF. Paired T-test (**p ≤ 0.01). (B) Correlation of TRIB3 expression with LPL, AP2 and PPARG (simple linear regression).

Supplementary Table 1 PCR protocol for genotyping

Process	Temperature	Time
Polymerase activation	95°C	5 min
Amplification 40x	95°C	30 sec
	65°C	30 sec
	72°C	30 sec
Inactivation	72°C	5 min

Supplementary Table 2 Primers for genotyping

Genotype	Primer sequence	Product size
WT	5’-CCGCGACGAATGAAAGGTTTA-3’	483 bp
	5’-AGACTCCGAGAGCTGCTCAGTTAGG-3’	
KO	5’-CCGCGACGAATGAAAGGTTTA-3’	381 bp
	5’-AAATGGCGTTACTTAAGCTAGCTTGC-3’	

[illegible]

Supplementary Table 3 Size intervals for LDL particle quantifications by ion mobility assay.

Lipoprotein Subclass Concentrations				
**Variable**	**Definition**	**Size intervals - Min and Max (Å)**		**Units**
HDL_3_2a_IM	High density lipoproteins 3 and 2a	76,50	105,00	nmol/L
HDL_2b_IM	High density lipoproteins 2b	105,00	145,00	nmol/L
Mid_Zone_IM	Midzone between HDL and LDL	145,00	180,00	nmol/L
LDL_IVc_IM	Low density lipoproteins IVc	180,00	190,00	nmol/L
LDL_IVb_IM	Low density lipoproteins IVb	190,00	199,00	nmol/L
LDL_IVa_IM	Low density lipoproteins IVa	199,00	204,90	nmol/L
LDL_IIIb_IM	Low density lipoproteins IIIb	204,90	208,20	nmol/L
LDL_IIIa_IM	Low density lipoproteins IIIa	208,20	214,10	nmol/L
LDL_IIb_IM	Low density lipoproteins IIb	214,10	220,00	nmol/L
LDL_IIa_IM	Low density lipoproteins IIa	220,00	224,60	nmol/L
LDL_I_IM	Low density lipoproteins I	224,60	233,30	nmol/L

Supplementary Table 4 Statistical analysis of lipid classes in Trib3 KD mature 3T3-L1 adipocytes vs wt cells. Analyzed by unpaired T-test with Welch's correction.

Species	P-value
Cer	0.0412 *
Cl	0.052
FA	0.4078
DG	0.0135 *
GLC	0.1151
PA	0.0395 *
PC	0.3002
PE	0.0412 *
PG	0.0691
PI	0.0811
PK	0.6326
PR	0.2553
PS	0.4665
SM	0.6358
SP	0.0686
ST	0.7110
TG	0.4429
Other	0.1358

Supplementary Table 5: Top altered and significant canonical pathways identified by IPA.

Ingenuity Canonical Pathways	Ratio	z-score	Down-regulated	No change	Up-regulated	No overlap
Triacylglycerol Degradation	0.116	-2.236	22/43 (51%)	0/43 (0%)	10/43 (23%)	11/43 (26%)
Protein Kinase A Signaling	0.016	-1.633	177/374 (47%)	0/374 (0%)	175/374 (47%)	22/374 (6%)
Heparan Sulfate Biosynthesis (Late Stages)	0.0806	-1.342	16/62 (26%)	0/62 (0%)	30/62 (48%)	16/62 (26%)
Heparan Sulfate Biosynthesis	0.0725	-1.342	17/69 (25%)	0/69 (0%)	35/69 (51%)	17/69 (25%)
Gα12/13 Signaling	0.0787	-1	57/127 (45%)	0/127 (0%)	61/127 (48%)	9/127 (7%)
PPAR Signaling	0.049	-1	49/102 (48%)	1/102 (1%)	41/102 (40%)	11/102 (11%)
Natural Killer Cell Signaling	0.0298	-1	70/168 (42%)	0/168 (0%)	84/168 (50%)	14/168 (8%)
Estrogen Receptor Signaling	0.0125	-1	166/321 (52%)	1/321 (0%)	135/321 (42%)	19/321 (6%)
RhoGDI Signaling	0.0636	-0.632	81/173 (47%)	0/173 (0%)	82/173 (47%)	10/173 (6%)
LXR/RXR Activation	0.0455	-0.447	50/110 (45%)	1/110 (1%)	38/110 (35%)	21/110 (19%)
Corticotropin Releasing Hormone Signaling	0.0355	-0.447	50/141 (35%)	0/141 (0%)	74/141 (52%)	17/141 (12%)
Role of NFAT in Cardiac Hypertrophy	0.0287	-0.447	91/209 (44%)	0/209 (0%)	105/209 (50%)	13/209 (6%)
SPINK1 Pancreatic Cancer Pathway	0.0741	0	15/54 (28%)	0/54 (0%)	13/54 (24%)	26/54 (48%)
ERK/MAPK Signaling	0.0324	0	95/185 (51%)	0/185 (0%)	76/185 (41%)	14/185 (8%)
Calcium Signaling	0.0308	0	76/195 (39%)	0/195 (0%)	95/195 (49%)	24/195 (12%)
Wnt/β-catenin Signaling	0.0298	0	69/168 (41%)	1/168 (1%)	83/168 (49%)	15/168 (9%)
Systemic Lupus Erythematosus In B Cell Signaling Pathway	0.0163	0	83/246 (34%)	1/246 (0%)	122/246 (50%)	40/246 (16%)
Opioid Signaling Pathway	0.0336	0.378	101/238 (42%)	0/238 (0%)	115/238 (48%)	22/238 (9%)
Hepatic Fibrosis Signaling Pathway	0.0198	0.378	154/354 (44%)	1/354 (0%)	172/354 (49%)	27/354 (8%)
IL-6 Signaling	0.0492	0.447	58/122 (48%)	1/122 (1%)	55/122 (45%)	8/122 (7%)
Gαi Signaling	0.0488	0.447	52/123 (42%)	0/123 (0%)	53/123 (43%)	18/123 (15%)
Relaxin Signaling	0.0411	0.447	65/146 (45%)	0/146 (0%)	73/146 (50%)	8/146 (5%)
Endocannabinoid Neuronal Synapse Pathway	0.0394	0.447	47/127 (37%)	0/127 (0%)	66/127 (52%)	14/127 (11%)
Endothelin-1 Signaling	0.0331	0.447	81/181 (45%)	0/181 (0%)	84/181 (46%)	16/181 (9%)
Senescence Pathway	0.0192	0.447	126/260 (48%)	0/260 (0%)	126/260 (48%)	8/260 (3%)
